# A decade of arboviral activity—Lessons learned from the trenches

**DOI:** 10.1371/journal.pntd.0005421

**Published:** 2017-04-20

**Authors:** Ann M. Powers, Stephen H. Waterman

**Affiliations:** 1 Division of Vector-Borne Diseases, CDC, Fort Collins, Colorado, United States of America; 2 Division of Vector-Borne Diseases, CDC, San Juan, Puerto Rico, United States of America; Baylor College of Medicine, Texas Children's Hospital, UNITED STATES

In the ten years during which *PLOS Neglected Tropical Diseases* has been publishing, it has become known as a critical journal for describing new and ground-breaking research in arbovirology. In its first year in existence, ten arboviral papers were published in this journal while last year, 157 arboviral papers were published here. In just the past decade, outbreaks of dengue, chikungunya, and most recently Zika have demonstrated how ever-increasing globalization has made mosquito-borne pathogens some of the most significant public health concerns known. During this time, even as these agents have become more prominent, significant advances have been made in understanding how to combat them.

Over ten years ago, the little known chikungunya virus (CHIKV), came to be known globally after an outbreak occurred in the resort island of La Reunion, leading to dozens of reported cases in European travelers and concern for how far it might move [1]. The virus spread to India where over 1 million cases were reported that year alone [2]. Both East Africa and India had previously experienced CHIKV activity, but the virus continued to spread over the next several years to subtropical latitudes and throughout the Americas where over 2.3 million infections have been reported to date. Just as CHIKV affected nearly every country in the Americas, Zika virus (ZIKAV), which was previously unknown to even most flavivirologists, was introduced into the Americas, leading to one of the largest global public health crises in years [3]. ZIKAV not only caused traditional vector-borne transmission febrile illness but was also found to be transmitted sexually and to cause devastating congenital abnormalities. During this same decade, while the high-profile CHIKV and ZIKAV outbreaks were in the news, outbreaks due to dengue viruses continued to occur with little fanfare. Dengue viruses still remain the leading cause of arboviral disease and are estimated to cause approximately 390 million infections per year [4].

Given this extensive pattern of arboviral epidemic activity, what has been learned by the public health and scientific communities that should be of use in future episodes? Primary is the need for sustained support for surveillance and research activities. Unfortunately, even though the arboviral community had spent significant time battling CHIKV, challenges with sustained support for arboviral surveillance and control efforts left this same group of health officials ill-prepared to handle yet another arboviral outbreak due to ZIKAV. Many of the efforts devoted to surveillance, diagnosis, messaging, and control are discontinued after an outbreak subsides because of a number of factors, including challenges with resources, competing public health priorities, and the sense that these issues aren’t really of concern any longer. Experience from the West Nile virus (WNV) activity in the Americas provides a good example of the need for sustained support. As WNV moved across the United States, over the course of several years, capacity to handle such emergence was built at the Centers for Disease Control and Prevention (CDC), public health laboratories, and academic research. However, erosion of this capacity included loss of trained epidemiologists, entomologists, and laboratorians combined with decreased funding for arboviral surveillance and diagnostics [5]. When WHO and CDC anticipated that CHIKV would reach the Americas and cause an outbreak of unprecedented scale, significant preparedness efforts were undertaken, including developing country-specific response plans, early epidemiological surveillance, laboratory training, and vector control planning [6]. However, the best plans can still be limited by lack of capacity, perceived need, or sufficient reagents and personnel. Even without considering the cost to individuals and quality of life of those affected, the economics of rebuilding versus maintaining capacity is staggering. Previous analyses demonstrated that the costs of responding to an outbreak in a delayed fashion could be as much at 346 times as high as the costs of response with an ongoing active surveillance program and early case detection [7]. This point has been clearly reiterated numerous times yet is still challenging to actually implement.

Preparedness for rapid outbreak response also includes the ability to accurately diagnose the cause of the epidemic. The fact that these recent globally spreading arboviruses cause similar clinical syndromes complicates their diagnosis, surveillance, and control efforts. More laboratory tests are needed, such as a recently developed polymerase chain reaction assay for all three viruses [8]. The Zika pandemic has also reinforced the need for more specific serologic tests for flaviviruses, as conventional immunoglobulin M (IgM) ELISA tests cannot in most cases reliably distinguish between Zika and dengue in endemic areas and when both viruses cocirculate, an increasingly common occurrence. Additionally, the utility of rapid point-of-care tests continues to be apparent, as most cases are never confirmed in a laboratory during large outbreaks. Improved diagnostics also can support enhanced surveillance platforms needed to test for multiple arboviruses in order to complement the traditional passive surveillance efforts of most countries. Enhanced surveillance will increase the likelihood of early detection of arboviruses that are new to a region.

Preparedness also necessitates having established integrated vector control. In particular, efforts for difficult urban vectors like *Aedes aegypti* with conventional or novel approaches should document effectiveness in reducing mosquito populations and disease incidence whenever possible. While mortality due to dengue can and has been reduced by good clinical care, further developing systems for continuing medical education and quality is necessary to reach the WHO goal of reducing dengue mortality by 50% [9] and to improve clinical diagnosis and care for all arboviral illnesses.

Lastly, even with advances in arboviral control programs, these efforts are likely to have the greatest impact in the context of a comprehensive strategy, which includes an unequivocal need for arboviral vaccines. Pathogens like CHIKV that were once limited to distinct geographic regions now maintain a nearly global distribution. Those in endemic areas would benefit significantly from a vaccine to avoid the months or years of extraordinary pain resulting from the chronic arthralgia associated with this virus. A vaccine against ZIKAV would prevent thousands of cases of congenital ZIKA syndrome; providing this to populations in endemic areas prior to reaching child-bearing age as well as to travelers to endemic areas could virtually eliminate the devastating birth defects associated with in utero infection. This is one area of success in global arboviral public health preparedness and partnerships. Finally, after over 20 years of research, vaccines against dengue viruses are a long-needed reality. The most recent reports have indicated that not only does over half of the world’s population continue to live in an area at risk of infection, but the incidence of dengue has increased by over 30-fold in the past several decades. This equates with nearly 100 million clinical infections per year with 2 million cases of severe disease and 21,000 deaths [10]. These numbers underscore that having a DENV vaccine available has been the very top arboviral disease control priority for years. One recombinant tetravalent live-attenuated dengue vaccine has been registered in a dozen or so countries for persons 9 years old into adult middle age, and WHO has recommended that countries consider its use in highly endemic areas. Two other tetravalent vaccines have recently begun Phase III clinical trials [11–13]. Hopefully, one or more dengue vaccines will eventually prove to be effective and safe in all ages and settings. The international coordination and cooperation that were needed to develop these vaccines and bring (to date) one vaccine to market represent the very finest in scientific achievement and demonstrate the importance and possibilities for success of global cooperation among public health, commercial, and academic partners in combating vector-borne diseases. We can only hope that this example will be a guide for how to continue to work to control these pathogens that know no boundaries.
